# Healthcare professionals’ and patients’ assessments of listed mobile health apps in China: a qualitative study

**DOI:** 10.3389/fpubh.2023.1220160

**Published:** 2023-09-14

**Authors:** PeiYu Liu, XueYun Li, Xiao Man Zhang

**Affiliations:** ^1^Department of Anesthesia and Operation Room, Nanjing Drum Tower Hospital, Nanjing, China; ^2^Department of Nursing, Xuzhou Medical University, Xuzhou, China

**Keywords:** patients, healthcare professionals, mobile health, quality, qualitative

## Abstract

**Background:**

In recent years, mobile health (mHealth) has gradually developed in China, and intelligent medicine has become an important research topic. However, there are still significant problems in mHealth applications (apps). Although healthcare professionals and patients are the main users, few studies have focused on their perceptions of the quality of mHealth apps.

**Objective:**

This study aimed to (1) understand the respective perceptions of healthcare professionals and patients regarding mHealth apps, (2) assess what barriers exist that influence the user experience, and (3) explore how to improve the quality of mHealth apps and the development of the mHealth market in China. The study aims to promote the standardization of mHealth apps and provide effective information for the improvement and development of mHealth apps in the future.

**Methods:**

Semistructured interviews with 9 patients and 14 healthcare professionals were conducted from January 2022 to April 2022 in the Affiliated Hospital of Xuzhou Medical University. The participants used mHealth apps for more than 3 months, including the “Good Mood” and “Peace and Safe Doctors” apps and apps developed by the hospital that were popular in China. Interview transcripts were analysed using thematic analysis.

**Results:**

The following five themes were extracted: different concerns, hidden medical dangers, distance and insecurity, barriers for older people, and having positive perceptions of mHealth apps. Healthcare professionals prioritized simplicity in regard to mHealth apps, whereas patients rated effectiveness as the most crucial factor. The study also revealed several problems with mHealth apps, including insufficient information about physician qualifications, inaccurate medical content, nonstandard treatment processes, and unclear accountability, which led to a sense of distance and insecurity among participants. Older individuals faced additional obstacles when using mHealth apps. Despite these issues, the participants remained optimistic about the future of mHealth app development.

**Conclusion:**

The utilization, advantages, and obstacles of mHealth applications for healthcare professionals and patients were explored through semistructured interviews. Despite the promising prospects for mHealth apps in China, numerous issues still need to be addressed. Enhancing the safety monitoring system and developing user-friendly mHealth apps for older adult patients are essential steps to bridge the gap between healthcare providers and patients.

## Introduction

### Background

In China, medical human resources are limited, and medical development is uneven. Mobile health (mHealth) is popular among Chinese residents as a convenient way to utilize equal medical resources (1). According to China’s iiMedia Group, the number of users of mHealth apps in China rose from 151 million to 298 million in 2015 ~ 2016, an increase of 50.6% over the previous year (2). The COVID-19 pandemic of recent years has also facilitated further advancements in mHealth apps. Mhealth apps provide equal medical resources to patients in remote areas and have good cost-effectiveness (3).

As smart medical devices, mHealth applications (apps) involve smart sensors, display screens, database storage and other multifunctions. These apps can monitor a user’s mental health, behavior, activity trajectory, and clinical data and remind users of correct behavior. Mhealth apps have been researched and developed in terms of pregnancy, smoking, asthma, pain, cancer, mental health, spirituality, and vision (4, 5).

Due to the continuous development of mHealth apps, the validity, acceptability, reliability, and quality of these apps need to be understood. To date, the evaluation of mHealth apps is mainly divided into clinical effectiveness (6–8), acceptability (9, 10), usability (9, 10), compliance (9), user experience (11, 12), and information quality (13, 14). Evaluation methods are also divided into three major categories: scales and questionnaires (15, 16), comparative experiments (6, 7), and qualitative interviews (9, 10). According to different evaluation methods, information and results from different perspectives can be obtained. Researchers often use comparative experiments to determine whether mHealth apps have improved regarding certain clinical indicators or cognition. Scales and questionnaires include the Mobile App Rating Scale (MARS) (17), the user version of the Mobile App Rating Scale (uMARS) (18), the System Usability Scale (SUS) (19), and self-designed questionnaires based on the Technology Acceptance Model (TAM) (20). The MARS and uMARS can be divided into four subscales (engagement, functionality, aesthetics, and information quality) and a qualitative scale that aims to examine users’ feelings during the use process (17, 18). The MARS and SUS have strong reliability and good internal and external validity, as recognized by most researchers (19, 21–23).

Qualitative interviews are used by researchers to understand interviewees’ internal and in-depth ideas. To investigate users’ evaluations of mHealth apps, Anderson (12) conducted qualitative interviews with individuals residing in university towns in Australia to gain insights into their experiences with and expectations of self-management apps. Serafica and colleagues (11) studied the experience of mHealth apps among people in rural Hawaii. In addition, in previous studies, qualitative interviews have been conducted to explore British COPD patients’ opinions on self-management apps (24), as well as to examine community health workers’ perspectives and experiences regarding mHealth apps in Brazil (25). However, it is unclear what experiences people in mainland China, which has a large population and many ethnicities, have with popular mHealth apps. In an era of rapid internet development, what do these individuals think of mHealth apps in China? What are the shortcomings that need to be improved, and what important information has been overlooked by developers? These issues are worth discussing.

In recent years, research on mHealth apps has gradually increased, and the types of mHealth apps for various diseases have also increased. Chinese researchers have utilized the MARS and Silberg Scale to assess the quality of various types of mobile health apps, including psychological (26), cardiovascular (27), sleep management (28), and postpartum depression apps (29), in terms of usability, effectiveness, and acceptability. Due to poor supervision and other issues, the quality of mHealth apps can be inconsistent, which can lead to decreased usage by users (30). Additionally, China’s vast population and diverse cultural differences among provinces and cities in different regions can make it difficult for mHealth apps to stand out in this crowded medical market (1).

In 1989, Davis proposed the TAM to explain users’ acceptance of information technology. The TAM suggests that the use of a system is determined by behavioral intentions, and behavioral intentions are determined by perceived usefulness and perceived ease of use (31). The diffusion of innovations theory discusses how new ideas, new things, and new products are accepted by the public (32). This theory divides the diffusion process into five stages (awareness, persuasion, decision, application, and determination) and emphasizes that mass communication can effectively provide new information. However, interpersonal communication is more effective in changing people’s attitudes and behaviors. According to these two theories, the experiences of healthcare professionals and patients with mHealth apps, as the main users, is particularly important. However, there are still gaps in the research on the experiences of healthcare professionals and patients in relation to mHealth apps in the Chinese healthcare market. To fill these knowledge gaps and address future software iterations, we conducted semistructured interviews with patients and healthcare professionals recruited from a tertiary grade A hospital. The purpose of this study was to (1) understand healthcare professionals’ and patients’ perceptions of the quality of mHealth apps; (2) evaluate the obstacles that affect users’ quality experiences; and (3) explore how to improve the quality of mHealth apps. The results are intended to promote the standardization of mHealth apps and provide effective information for the improvement and development of mHealth apps in the future.

## Methods

### Design and setting

In this study, semistructured interviews were conducted at the Affiliated Hospital of Xuzhou Medical University, which is a general hospital in Xuzhou, Jiangsu Province, China. The Affiliated Hospital of Xuzhou Medical University was founded in 1897 and has a history of 123 years. In the first half of 2021, nearly 1.4 million patients received outpatient and emergency services at this hospital, with nearly 90,000 discharged patients. Although Xuzhou belongs to Jiangsu Province, as an important transportation hub city, it has close ties with Shandong, Anhui, Henan and other provinces. The hospital treats many patients. Xuzhou also has a long history of more than 6,000 years of civilization and 2,600 years of history, and the population is more than 8 million. The hospital has developed a series of medical treatment and management systems. Healthcare professionals working in this hospital have access to mHealth apps and use them frequently.

### Participants

By using purposive sampling and the principle of maximum differentiation, patients who visited this hospital and clinical frontline healthcare professionals who worked in this hospital from January to April 2022 were selected as participants for this study. The selection criteria for patients were as follows: patients who know what are mHealth apps; those who had used at least one mHealth app; those who had used the apps for at least 3 months; those aged ≥18 years; those with clear awareness and thinking that allowed good communication; and those who volunteered to participate in this study. The selection criteria for healthcare professionals were as follows: healthcare professionals who had worked on the clinical front line for ≥3 years; those who had used the apps for at least 3 months; those with good communication skills; and those who volunteered to participate in this study.

According to a literature analysis (33–35), the evaluation period of the majority of mHealth app intervention studies can be roughly divided into pre-and postintervention periods, with four treatment cycles (12 weeks) before and after the intervention. Based on the 21-day effect of behavioral development and a literature analysis, this study set a target of the use of mHealth apps for a minimum of 3 months to ensure that participants had a thorough understanding of the mHealth apps they used. The sample size was determined based on data saturation, which occurs when the information provided by interviewees becomes repetitive and no new topics emerge.

### Interview outline

The study adopted a descriptive research method, utilizing interviews to gain a comprehensive understanding of the quality evaluation of mHealth apps from the perspectives of both patients and healthcare professionals. In accordance with the study’s objectives, two nurses working in the pain department were chosen for preinterviews after a thorough review of the relevant literature and consultations with two members of the research team. According to the preinterview situation, an expert with rich research experience was consulted, and discussions and revisions were conducted to determine the final interview outline (Table 1).

**Table 1:** Qualitative semi structured evaluation guide for healthcare professionals and patients.

Serial number	Healthcare	Patients
①	Have you used any mHealth apps (if the mHealth app has a patient version)? Would you recommend it to your friends?	
②	What do you think about the development of mHealth apps in the future?	
③	For the mHealth app that you have used, what do you think are its shortcomings? What else do you think needs to be improved?	
④	If you were to evaluate an mHealth app, what aspects/perspectives would you use? What indicators do you think are the most important for evaluation?	

### Data collection

Researchers introduced the purpose and methods of this study to patients at the outpatient department. After obtaining their consent to participate, the researchers asked the patients if they knew what mHealth apps were. If the patients could name one or more apps, the researchers proceeded to the next question. If not, the patients were terminated from the study and did not proceed with the enrolment process. This approach ensured that only patients who had some knowledge of mHealth apps were included in the study. Then, researchers inquired about whether they had previously used a mHealth app, what the name of the app was, how long they had been using it, and how often they used it. The selection of healthcare professionals was based on communication with department managers, who selected individuals based on their work experience. Then, researchers asked questions about whether they used mHealth apps in their work, the amount of time spent using them, and the frequency of use per day. Based on these questions, individuals were deemed eligible for inclusion in the study if they met the inclusion criteria; otherwise, they were excluded.

Due to the long wait times of approximately 1–2 h, interviews with patients were conducted during the patient’s wait time. After consulting with the department head nurse and doctors, the interviews with healthcare professionals were scheduled in the afternoon of a workday, with each healthcare professional invited and allotted 1 h of sufficient time for the interview. All interviews took place in a quiet classroom in the hospital. One-on-one, face-to-face interviews were conducted by a qualitative research-trained researcher. The entire interview process was recorded with a smartphone, and the key content and nonverbal content were recorded on paper. The interviews lasted approximately 30 min. When the researcher did not understand an answer, she repeated it to the participant to determine whether the meaning of the expression was accurate.

### Data analysis

Within 24 h of the interview, the researcher transcribed the interview contents into written materials, and then another team member checked and ensured the accuracy of the transcript. After the transcription was completed, the interview subjects determined whether the transcription content accurately expressed their meaning or whether it needed to be modified. We used the standard method of thematic analysis to analyse the transcripts. Two researchers analysed the data independently. First, the researchers read the transcript in depth several times until they felt that they understood what the participants had said. Second, open coding was initially started line by line. Significant fragments were extracted, which were identified as “quotes,” and assigned “codes.” Third, the meaning was formulated for the codes, which were then organized into themes. Finally, the themes were further integrated into descriptions.

### Ethical considerations

Ethical approval was obtained from the Affiliated Hospital of Xuzhou Medical University Ethics Review Board (Reference Number: XYFY2019-KL018). Information about the study was explained to all participants before the interviews, including information on research confidentiality and the participants were advised regarding their ability to withdraw from the study at any time during the interview. Participants were asked if they agreed to record the entire interview; if they did not want the interview recorded, a change was made immediately to manual recording. Finally, the participants signed written informed consent forms and volunteered to participate.

## Results

### Participant characteristics

A total of 14 healthcare professionals and 9 patients were interviewed. Their ages ranged from 24 to 57 years. The healthcare professionals had all worked in clinical first-line therapy for more than 3 years (Table 2). The patients were all from the area surrounding Xuzhou and were engaged in different industries, such as decoration design, teaching, and homemaking, and different departments (Table 3). The mHealth apps used by the participants are presented in Appendix 1. These mHealth apps are frequently used in China and are popular based on the number of downloads.

**Table 2:** Characteristics of the interviewed healthcare professionals.

Sociodemographics	Statistics
Profession, *n*	
Nurse	10
Physician	4
Age (years)	
Range	25
Mean	34.57
SD	7.07
Sex	
Male	5
Female	9
Professional experience (years)	
<5 years	2
5–10 years	7
10–15 years	3
>15 years	2
Frequency of mHealth app usage	
<3 times/day	2
>3 times/day	12

**Table 3:** Characteristics of the interviewed patients.

Sociodemographics	Statistics
Age (years)	
Range	33
Mean	35.88
SD	10.57
Sex	
Male	3
Female	6
Educational Level	
Junior high school	2
High school diploma or equivalent	3
College	4
Residence	
Rural	5
Urban	4
Frequency of mHealth app usage	
<3 times/day	7
>3 times/day	2

### Themes and subthemes

A total of five themes were identified: (1) different concerns; (2) hidden medical dangers; (3) distance and insecurity; (4) barriers for older people; and (5) having positive perceptions of mHealth apps.

### Theme 1: Different concerns

In the interviews, 7 (7/14, 50%) healthcare professionals considered easy operation to be the focus of the evaluation (Table 4). A nurse shared her feelings about mHealth during her work:

**Table 4:** Focus of mHealth app quality assessments by healthcare professionals and patients.

	Healthcare professionals		Patients	
Encoding	Number of persons	Percentage	Number of persons	Percentage
Easy operation	7	50%	2	22%
Effectiveness	2	14%	6	67%
Function	2	14%	2	22%
Accessibility	3	21%	1	11%
Security	6	43%	5	56%
Payment	2	14%	2	22%

*Now with the electrocardiograph machine, it is a relatively easy operation. One key or two keys can be used; it is not complex. For example, for the current electrocardiogram, you press a key. If an app requires data to be filled in, it will be a challenge for the patients.* (M3)

Another nurse shared that her workload was increased by cumbersome mHealth operations:

*A lot of patients ask us to help them, which indirectly increases our workload. We always have miscellaneous tasks every day.* (M4)

Furthermore, 6 (6/9, 67%) patients agreed that effectiveness was the most important part of their evaluation of mHealth apps. According to one patient,

*The most important thing, I think, is that it's helpful, effective, and beneficial regarding my illness. If the software is not helping me, why do I want to use it?* (P4)

## Theme 2: Hidden medical dangers

### Subtheme 1: Insufficient information about qualifications

Four (4/9, 44%) interviewed patients said that the qualifications of the healthcare professionals were not included on the platform, and they could not objectively judge the professionals’ medical levels. A patient described the awkwardness of using the app to select an obstetrics and gynaecology professional because of the unknown qualifications of the healthcare professionals in the mHealth app:

*I selected a gynaecology professional on it; maybe I didn't see it clearly. I thought the doctor was a woman when I looked at the doctor's name at first. Then, I looked again—a man. I can't see a male doctor. I didn't want to…I didn't want him to check.* (P1)

Another patient believed that the authenticity of mHealth apps would be confusing if they did not detail the qualifications of healthcare professionals:

*Every doctor's skill is really important. Now there are only two things to say about the software, such as what the doctor is, but not exactly. Of course, doctors with high grades are certainly relatively experienced. Users can also evaluate these doctors, not just show good evaluations. Some bad evaluations are blocked in some software, so we do not trust the authenticity of the software.* (P9)

### Subtheme 2: Inaccurate medical content

Five (5/9, 56%) interviewed patients said that the health information provided by the mHealth apps did not have a detailed source description, and they could not judge the scientific nature and authenticity of the information. A mother shared her doubts about the information provided on a mother-to-child mHealth app during her pregnancy:

*It's a bizarre statement that pregnant women can’t eat this and that on this app. In the past, pregnant women could eat anything that a normal person could eat. I'm generally selective about what's on this app.* (P2)

A patient who came for a medical examination said that the low authenticity of the content provided by the mHealth app would affect his compliance:

*If the authenticity is low, I don't believe what it is providing, so my compliance is low, and even if it’s right, I' m still reluctant to follow it.* (P4)

Furthermore, a patient said that due to poor authenticity, he selectively used mHealth apps; if he had problems, he still sought a doctor for the first time:

*Some of the online content is right or wrong … even though I know that some health care knowledge in the app is good, I still mainly seek a doctor's advice.* (P9)

### Subtheme 3: Nonstandard treatment processes

A standardized medical treatment process can effectively reduce the incidence of medical accidents and disputes (36). Standardized processes not only reduce human injury for patients but also protect healthcare professionals. Two doctors (2/14, 14%) reported that the medical procedures of an mHealth app were not standardized, which could lead to medical errors:

*The medical software needs to be more rigorous to meet the basic requirements of the National Health Commission and to ensure safety. All processes and operations should be scientific, regular and secure. It's a big taboo to prescribe prescription drugs and diagnose patients. We can' t afford the consequences of misdiagnosis. We can't tell if there's a problem. We can only give patients some medical advice or health measures they can take.* (M7)

*I personally believe that the regulation of smartphone medical software is relatively loose. The entire process is not perfect.* (M8)

### Subtheme 4: Unclear accountability

According to two participants (2/23, 9%), the responsibility of mHealth apps is unclear, there are hidden dangers, and it is difficult to determine responsibility.

*I don't know who I can look for if there's something wrong with pills.* (P5)

*Once the mistake and the inevitable consequences have taken place, who should be responsible for them? This cannot be determined clearly. There are also problems in the hospital that are unclear. Now there are too many doctor–patient disputes.* (M7)

## Theme 3: Distance and insecurity

### Subtheme 1: Blunt expression

The counseling function of mHealth apps is mainly in the form of text input and output (37), and healthcare professionals cannot observe the body language of patients. In addition, patients are prone to doubt the responses of healthcare professionals, which produces a sense of distance and insecurity. Four participants (4/23, 17%) said mHealth apps display too much text, less vivid dynamic graphics and less videos and are difficult to understand. A nurse with rich clinical experience believed that presentation can not only close the distance between nurses and patients and improve patient compliance but also meet the needs of patients at all cultural levels:

*From the patients' point of view, if you recommend mHealth apps well, they’ll see more. Like the rich form I've just said: if every health education app has video education, it is convenient for the patient. You'll be able to close the distance from the patient, and improve their compliance, cooperation with treatment or rehabilitation. In addition, there are also cultural levels and regional differences. For example, a person with a high level of education, you go to tell them the information is not as good as their own. However, patients with a low educational level or patients from some rural regions want you to say it, write it down, rather than taking their own initiative to learn.* (M4)

In addition, some nurses suggested that more dynamic videos could be made:

There can be some health education videos … dynamic. (M12)

A patient wanted to be provided with video calls and face-to-face communication:

*How did the doctor judge which situation I was in? It would be best to have a video to communicate. In addition, I wasn't very good at typing. I feel a little bit relieved to see the doctor himself in the video, at least feel zero distance.* (P9)

### Subtheme 2: Generating medical virtuality

With the development of networks and technology, intelligent virtual agents have permeated various fields, such as medicine, teaching, work and life, promoting the development of society (38). However, because of the particularity of the target population of mHealth apps, they often cause insecurity for users:

*Like apps, many times the consulting doctor is not local. If a problem emerges, I do not where I can go to find them.* (P4)

Another patient said the medical software led to a sense of distance:

*I have a distant feeling in the app that I'm not talking to real people and that sometimes it's the robot that automatically replies. It's not as good as talking to a doctor in person. I can also understand the doctor's level and the medical level of the hospital.* (P2)

### Subtheme 3: Lack of humanistic care

Mhealth apps can expand the medical network, connect patients from multiple fields, and share medical resources equally. Although communication can increase peer support, humanistic care and cross-cultural differences are ignored. Users receive cold and mechanized responses. A patient believed he could receive warm care from paramedics in the hospital, but the app only responded automatically:

*I received polite replies (on the app), but they were cold and official. Although doctors also have a bad attitude in the hospital, there are doctors and nurses in the hospital every day to check in and ask you how you slept last night. After you leave the hospital, the nurse will follow up on your recent situation. However, it's over when you' re done consulting on the app, and no one cares about your follow-up.* (P5)

*I don't feel like anyone cares about me. A smart device can't do what doctors and nurses do, and no one cares what danger will happen to you. In addition, the speed of the response of apps is too slow, which made me feel I am not important ….* (P7)

## Theme 4: Barriers for older people

### Subtheme 1: Terminology and professionalization

The main difference between mHealth apps and other apps lies in the purpose of mHealth app use and the particularity of the population (25). Medical terminology and medical professionalism are also unique to mHealth apps. Due to degenerative changes in the brain and organs of older people, their ability to perceive, think, remember and understand is weakened. They may feel a certain degree of psychological resistance before using medical apps, thus reducing their desire to use these apps (39). A doctor shared his experience in recommending medical software to older patients:

*When I recommended the ‘Good Mood’ app to the patients, I found that the older patients rejected it directly and said that they did not understand it when I told them they would have to use a smartphone.* (M7)

Because of late contact with smart devices and poor understanding in China, older people tend to resist mHealth apps:

*Due to the particularity of the work, we connect the older patients or children. Older patients have a poor understanding, and their generation has a low level of education and medical knowledge, even with oral explanations. They may not understand, let alone use the app.* (M3)

### Subtheme 2: Unfriendly technology and design

A small font size and cumbersome operations seriously affect older patients’ experiences of mHealth apps, which are also common problems with smartphone apps. A 56-year-old patient had several operational problems during the use of registered services:

*Apps have to be downloaded and constantly remind you to update, which is especially complex. I have to register a service repeatedly. My patience wears out. I have no confidence in myself.* (P9)

Healthcare professionals and individuals who cohabitate with older relatives frequently encounter senior citizens who lack proficiency in utilizing mHealth apps. Older people come to seek their help. However, they said that this indirectly increases their burden:

*It doesn't feel good to use it because when an older patient comes, the process of linking a card and a bank card can be slightly more cumbersome. He will not do it, so we help him.* (M2)

*I have two old people in my family. Some software is said to be used for elderly individuals, but older people cannot see clearly and do not know how to use it. Well, we as their children will help them, but we also have our own things that keep us busy.* (P2)

## Theme 5: Having positive perceptions of mHealth apps

In the interviews, the healthcare professionals and patients had a positive attitude as the main users of mHealth apps. While participants criticized their drawbacks, they looked forward to future mHealth apps to bring new changes to society. For example, although 23 participants noted that the current mHealth apps have many quality problems that seriously affect their use experience, they answered positively when asked whether they would recommend these apps to others. They believed that the future medical model would reach a new platform if developers could effectively improve the quality of mHealth apps:

*As young people grow older, they will prefer to use smartphone apps rather than go to the community to do these things. So I suggest that when the community can promote this, and then young people use these apps for a long time, your medical information is equivalent to filing in this community, which is actually good for the later development of the community and can reduce the waste of medical resources in big hospitals.* (M7)

## Discussion

### Principal findings

This study was conducted during the implementation of lockdown regulations in China, a time when the use of mHealth or telemedicine provided a novel approach to patient care. The purpose of this study was to examine the experiences of healthcare professionals and patients who used mHealth apps and to identify the challenges they faced while using these apps. The primary findings indicated that there are several issues related to the quality of mHealth apps, although users still recognize the benefits of these apps. This may be attributed to the limited access to hospital-based medical care during the COVID-19 lockdown period in China, where patients relied on mHealth apps to receive timely and effective medical attention from healthcare professionals.

The participants were drawn from a range of clinical areas, including pain management, oncology, psychiatry, thymic surgery, general surgery, obstetrics and gynaecology, and physical examination centres. Based on their own usage experiences, the participants demonstrated the most frequently utilized features, such as appointment scheduling and registration, the dissemination of health information, and online consultations. These findings are in line with the results reported by Anderson (12) and Wu (40). MHealth apps can provide various benefits to patients, such as saving time, enhancing the efficiency of medical treatment, and reducing discomfort and anxiety. During the period when lockdown regulations were implemented in China to combat the COVID-19 pandemic, patients demonstrated a preference for using mHealth apps for appointment scheduling or consultation purposes, thereby reducing the time and opportunities spent in social contact with others.

Although the nine patients had different types of diseases, the interviews did not reveal any differences in their experiences using mHealth apps based on their specific medical conditions. It is possible that the participants had relatively mild or subhealth conditions, which may have influenced the results. However, according to Wang’s systematic review (41), the effectiveness of mHealth functions can vary depending on the specific type of disease being treated. A meta-analysis (42) showed that mHealth apps had no significant effect on drug compliance for patients with various chronic diseases. Further research is needed to determine whether different types of chronic diseases have varying effects on each function and module of mHealth apps.

It was found that Chinese patients prioritized the effectiveness of mHealth apps in managing their illnesses, whereas Chinese healthcare professionals favored apps with user-friendly interfaces and optimized designs. However, Jiang et al. found that healthcare professionals were primarily interested in the economic benefits of mHealth apps in reducing healthcare costs (43). Xiang’s study found that Chinese healthcare professionals prioritized the ability of artificial intelligence to reduce repetitive workload, and nonhealthcare personnel valued efficiency in diagnosis and treatment. Both groups of participants reported that artificial intelligence does not pose any safety risks (44). In contrast, this study found that both Chinese healthcare professionals and patients perceive mHealth as having safety issues. Furthermore, we propose that the government needs to establish strict procedural guidelines for the development and regulation of mHealth. These findings suggest that there is a need for more comprehensive research on the impact of mHealth on patient safety, as well as for the development of appropriate regulatory frameworks to ensure the safe and effective use of this technology (45). Generally, this study highlighted the importance of considering the perspectives of different stakeholders when designing mHealth apps.

During the interviews, healthcare professionals expressed concerns about the burden placed on their work by older patients who are unable to use mHealth apps. With the implementation of China’s three-child policy, the number of female clinical doctors and nurses on maternity leave has increased, resulting in healthcare professionals having to assist patients with basic tasks and teach them how to use mHealth apps. This has undoubtedly increased the workload of healthcare professionals. These findings highlight the need for medical institutions to employ dedicated internet staff or information nurses to address these issues. This study provides valuable insights into the underresearched area of user perspectives.

Healthcare professionals also faced challenges when assisting older patients who lacked necessary information such as call numbers and personal identification information required for verification. This issue may be related to the lower education levels of contemporary older individuals (46) or the larger number of hospitalized patients in nearby rural areas. These findings are consistent with those of Raghunathan’s study (47). Older individuals aged over 80 years were less willing to use mHealth apps, and those with higher levels of education were more accepting of these apps than those with lower levels of education (48). Therefore, mHealth app designs should not only cater to young and middle-aged individuals but also focus on the needs of older users.

It was found that the interviewed patients perceived mHealth apps as lacking in care, which could potentially strain the doctor–patient relationship. Furthermore, healthcare professionals in China face challenges due to heavy workloads and low wages (49, 50). Simpkin et al. (51) explored the perspectives of medical students on intelligent healthcare and gained insights into how to integrate technology effectively and consciously into the medical field to enhance empathy and compassionate care. To address the concerns raised by patients, a feedback function could be added to the platform, mHealth apps could be developed with a patient-centred approach (48), and humanistic theory could be applied to intelligent healthcare.

The quality issues of mHealth have also been emphasized by international organizations and researchers from other countries. To assist developers in evaluating the quality of apps, several tools have been developed (52). A systematic review of 87 studies published through 2018 revealed that researchers developed 48 different rating scales for evaluating the availability and quality of mHealth apps (52). These rating scales primarily focused on content quality and usability. This study uses open-ended questions to overcome these rating frameworks. The findings indicated that there were quality issues in mHealth apps from various aspects, including security risks, technical issues, design, and humanistic concerns. International organizations considered the entire quality regulatory issue from a larger perspective, accounting for the interests of multiple parties (53). They believed that improving the efficiency of mHealth regulation should emphasize process transparency rather than imposing accountability (54). This study concluded that healthcare professionals and patients considered the need for more standardized accountability to clarify their own work responsibilities or better protect their individual rights.

## Implications for future research and clinical practice

The participants of this study identified various barriers in the areas of process, regulation, technology and humanistic. Additionally, they found that mHealth apps increase workloads for both healthcare professionals and individuals who cohabitate with older relatives to a certain extent. Drawing on Shachak’s research findings (55), future research directions in health information technology implementation should not only focus on users’ social psychological issues and usage behavior but also consider technology, processes, contexts, and users as a dynamic interactive system. In a multicultural, multireligious, and multiethnic Asian country with a predominantly older population, medical humanities can help patients feel respected and cared for. China is a country that values family-oriented traditions, and the loneliness experienced by older individuals who live alone can easily lead to mental illness. MHealth apps can provide these individuals with more medical and social support. Future studies should focus on continuously improving the quality of mHealth apps while considering how to better integrate humanistic care with scientific and technological advancements.

### Strengths and limitations

The primary strength of our study lies in the use of qualitative methods to explore the experiences of healthcare professionals and patients with mHealth apps, as well as the factors influencing the continued use of mHealth apps by users in the Chinese healthcare market. However, a major limitation of our study is the small sample size, limited to individuals from XuZhou, the results are not representative of the whole of China, and the fact that only the views of healthcare professionals and young patients with older patients around them were considered. Although the selected mHealth apps have a high download frequency in the market, these apps could not represent all mHealth apps in China. To address this limitation in future research, we can expand the sample size and increase the diversity of the sample by including more technicians and related personnel in drug enterprises. Additionally, importantly, the participants in our study were homogeneous in terms of ethnicity; therefore, our findings may not accurately represent minority groups outside this demographic background.

## Conclusion

This study aimed to explore the perceptions of individuals regarding mHealth apps in China. Healthcare professionals and patients expressed optimism about the potential of mHealth apps but also highlighted several concerns and potential risks associated with their quality. Thus, it is crucial to enhance the safety and regulatory frameworks surrounding mHealth apps and to develop user-friendly mHealth apps specifically designed for older patients, which will help bridge the gap between healthcare professionals and patients.

## Data availability statement

The original contributions presented in the study are included in the article/Supplementary material, further inquiries can be directed to the corresponding author.

## Author contributions

PL contributed to the study concept and design. PL and XL contributed to the acquisition of data, data analysis, interpretation of data, and drafting of the manuscript. XZ contributed to the interpretation of data, and critical revision of the manuscript and final approval of the manuscript. All authors contributed to manuscript revision, read, and approved the submitted version.

## Funding

This study was funded by the University Research Board of Xuzhou Medical University (grant number 2018KJ07) and the Xuzhou Science and Technology Bureau (grant number KC19223).

## Conflict of interest

The authors declare that the research was conducted in the absence of any commercial or financial relationships that could be construed as a potential conflict of interest.

## Publisher’s note

All claims expressed in this article are solely those of the authors and do not necessarily represent those of their affiliated organizations, or those of the publisher, the editors and the reviewers. Any product that may be evaluated in this article, or claim that may be made by its manufacturer, is not guaranteed or endorsed by the publisher.

## Abbreviations

mHealth: Mobile Health
App: Application
MARS: Mobile App Rating Scale
uMARS: User Version of the Mobile App Rating Scale
SUS: System Usability Scale

## Appendix 1

MHealth apps used by participants and their functions and basic information.
