# Supplementary material for: Healthcare professionals’ and patients’ assessments of listed mobile health apps in China: a qualitative study
Source: Front Public Health. 2023 Sep 14;11:1220160. doi: 10.3389/fpubh.2023.1220160 (PMC10538635; doi:10.3389/fpubh.2023.1220160)
Supplement: Supplementary file 1 [file Table_1.pdf]

Mhealth apps used by participants and their functions and basic information.

App	Main feature	Times of installations	Score in app store	Year of developm ent
Peace and Good Doctor	1. Online shopping for medical supplies<br>2. Online consultation and communication with doctors from different areas and hospitals<br>3. Health knowledge promotion and health report interpretation<br>4. Making appointments with doctors	200 million	2.9	5
Dr. Clove	1. Online consultation and communication with doctors<br>2. Inquiring about medical information<br>3. Daily health care knowledge promotion	22.46 million	3.1	6
Good Mood	1. Online consultation and communication with doctor<br>2. Mental and mood scale self-assessment<br>3. Interpretation of related diseases such as psychology and spirituality<br>4. Online shopping for medical	39 thousand	5.0	7

	supplies<br>5. Reserving Central and Mental Genetic Testing Project			
Sugar Nurses	1. Recording and monitoring daily blood glucose<br>2. Analysis of blood glucose fluctuations<br>3. Developing a blood sugar control plan<br>4. Recording medication plan	1.19 million	4.6	3
Good Doctor	1. Online consultation and communication with doctor<br>2. Making an appointment with a doctor<br>3. Patient information exchange platform<br>4. Health knowledge promotion and health report interpretation	100 million	4.7	7

Spring Rain Doctor	1. Online shopping for medical supplies<br>2. Online consultation and communication with doctor<br>3. Querying related information such as hospital information and the medical level of medical staff in departments<br>4. Health knowledge promotion<br>5. Scheduling a physical examination	25.21 million	4.2	9
Pain Housekeeper[a]	1. Pain self-assessment<br>2. Self-evaluation of physical and psychological symptoms questionnaire<br>3. Analysis of pain value fluctuation<br>4. Online consultation and communication with doctor<br>5. Medical nurses remotely monitor the health of patients and provide guidance and suggestions<br>6. Promoting knowledge on the correct handling of acute and chronic pain	/	/	4

Palm Hospital[b]	1. Hospital test report transmission<br>2. Patient disease recording and fluctuation detection<br>3. Online medical bill payment	/	/	6
Beautiful Shaddock	1. Records of menstrual period time, amount, colour, pain degree, etc<br>2. Reminder of safety period and ovulation period<br>3. Sharing of health knowledge about menstruation and pregnancy<br>4. Female gynaecological diseases and sharing pregnancy experiences	300 million	3.6	7
Alipay[c]	1. Online shopping for medical supplies<br>2. Online consultation and communication with doctor<br>3. Health report interpretation<br>4. Making an appointment with a doctor	4.7 billion	3.5	17

Mother's Net	1. Introducing precautions for pregnancy preparation, pregnancy and postpartum health<br>2. Pregnancy preparation, birth check, postpartum experience sharing<br>3. Records of menstrual period time, mood, body temperature<br>4. Purchasing pregnancy essentials and health products online	100 million	4.0	16
Mother Group	1. Pregnancy self-test and hospital testing instruction<br>2. Introduce precautions for pregnancy preparation, pregnancy and postpartum health<br>3. Pregnancy preparation, birth check, postpartum experience sharing	18.73 million	4.6	18

The number of installations and score of the app store are both from the app store.

a. "Pain Housekeeper" was developed by the research team and is used in the pain department and oncology department. There is no related software introduction in the app store.

b. "Palm Hospital" was developed by the hospital and is used in all departments of the hospital. There is no related software in the app store.

c. “Alipay” is third-party payment software developed by Ali Group that has been widely used in China. A medical convenience function was implemented in Alipay's living city module.

The purpose of the participants using the mhealth apps and the departments interviewed by the participants

App	Purpose of use by participants	Department of participants
Peace and Good Doctor	1. Reducing the waiting time for consultation and making appointments directly<br>2. Getting medical information	Department of General Surgery
Dr. Clove	1. Reducing the waiting time for consultation	Department of Chinese Medicine
Good Mood	1. Consulting and buying drugs online<br>2. Self-assessment of mental symptoms<br>3. Understanding the patient's condition	Department of Psychiatry and Psychology
Sugar Nurses	1. Understanding blood sugar fluctuations and adjusting diet	Diabetes
Good Doctor	1. Reducing the waiting time for consultation and making appointments directly	Department of Thymus
Spring Rain Doctor	1. Making an appointment for a physical examination to reduce queues<br>2. Interpretation of the medical examination sheet to understand the physical condition	Medical Examination Centre
Pain Housekeeper	1. Keeping abreast of patient pain fluctuations and providing remote guidance for special situations<br>2. Understanding the patient's medication status and adjusting the plan	Department of Pain and Oncology<br>Cancer
Palm Hospital	1. Obtaining the patient's body report data<br>2. Paying on time to avoid delay in treatment	Department of General Surgery and Respiratory

Beautiful Shaddock	1. Recording the menstrual period and understand whether the monthly menstrual period is normal<br>2. Looking at other people's shared experience of similar gynaecological diseases	Department of Obstetrics and Gynaecology
Alipay	1. Scheduling a physical examination	Medical Examination Centre
Mother's Net	1. Learning about diet and exercise during pregnancy<br>2. Understanding the check items that must be done during pregnancy and why this check is needed	Department of Obstetrics and Gynaecology
Mother Group	1. Learning to self-check at home<br>2. Exchanging experiences with other expectant mothers	Department of Obstetrics and Gynaecology
